# Effects of parental mental health and family environment on impulsivity in preadolescents: a longitudinal ABCD study^®^

**DOI:** 10.3389/fnbeh.2023.1213894

**Published:** 2023-10-24

**Authors:** Nioud Mulugeta Gebru, Priscila Dib Goncalves, Rick A. Cruz, Wesley K. Thompson, Nicholas Allegair, Alexandra Potter, Hugh Garavan, Julie Dumas, Robert F. Leeman, Micah Johnson

**Affiliations:** ^1^Center for Alcohol and Addiction Studies, Department of Behavioral and Social Sciences, Brown University School of Public Health, Providence, RI, United States; ^2^Department of Health Education and Behavior, University of Florida, Gainesville, FL, United States; ^3^Department of Epidemiology, Columbia University Mailman School of Public Health, New York, NY, United States; ^4^Department of Psychology, Arizona State University, Tempe, AZ, United States; ^5^Center for Population Neuroscience and Genetics, Laureate Institute for Brain Institute, Tulsa, OK, United States; ^6^Department of Psychiatry, University of Vermont, Burlington, VT, United States; ^7^Department of Health Sciences, Northeastern University, Boston, MA, United States; ^8^The Department of Mental Health Law and Policy, University of South Florida, Tampa, FL, United States

**Keywords:** family conflict, parental depression, substance use, impulsive, social context, youths

## Abstract

**Introduction:**

Impulsivity is a known risk factor for the development of substance use disorders and other psychiatric conditions that is influenced by both genetics and environment. Although research has linked parental mental health to children’s impulsivity, potential mediators of this relationship remain understudied. The current investigation leverages the large national Adolescent Brain Cognitive Development (ABCD) Study to assess the mediating role of family conflict – an important social context for youth development – in the relationship between parental mental health and youth impulsivity.

**Methods:**

Data were from the first three annual waves of the ABCD study (Baseline *N* = 11,876 children, *M*_age_ = 9.9 years; 48% female; 52% White). Parental mental health conditions were self-reported internalizing, externalizing, and total problems. Youth completed the family conflict scale, and Urgency, Planning (lack of), Perseverance (lack of), Sensation Seeking, and Positive Urgency (UPPS-P) scale to measure impulsivity. To determine if within-family change in conflict from baseline to year 1 explained changes in the strength of relations between baseline parental mental health and year 2 youth impulsivity, longitudinal causal mediation analyses were conducted, controlling for demographic factors (i.e., age, sex, race, household income, parental education, marital status), as well as baseline levels of family conflict and outcomes. Separate mediation models were run for each mental health condition and each UPPS-P subscale.

**Results:**

Above and beyond bivariate relations, longitudinal mediation models, which included covariates, showed family conflict significantly (*p*s < 0.001) mediated relations between all three parental mental health conditions and all but one (i.e., sensation seeking) UPPS-P subscales. The proportion mediated through family conflict for internalizing problems and total problems on facets of impulsivity (except sensation seeking) ranged from 9% (for lack of perseverance) to 17% (for lack of planning). Proportion mediated via family conflict for externalizing problems on youth’s impulsivity (except sensation seeking) was slightly higher, ranging between 13% (lack of perseverance) to 21% (lack of planning).

**Discussion:**

Family conflict may be an important intergenerational factor linking parental mental health and youth’s impulsivity. Addressing parental mental health and family conflict may help curb increased impulsivity in youth, and in turn reduce adolescent substance use disorders.

## 1. Introduction

Impulsivity is a multi-faceted construct characterized by an overall rapid response to stimuli (Moeller et al., 2001; Brewer and Potenza, 2008; Leeman et al., 2019). Aspects of impulsivity include lack of planning, lack of perseverance, sensation seeking, as well as positive and negative urgency (Whiteside and Lynam, 2001). Impulsivity’s role in the initiation and acceleration of substance use among adolescents has received significant attention in the literature (Quinn and Harden, 2013; Vergés et al., 2019; Waddell et al., 2022). Substance use is common among adolescents, with nearly a third of 12th graders (typically 17–18 years old; 32.6%) reporting any illicit drug use in the past year and over half (51.9%) reporting alcohol use in the past year (Johnston et al., 2022). Adolescent substance use is associated with negative effects on the brain and cognition, poor academic performance, and an increased likelihood of developing a substance use disorder in the future (Warner and White, 2003; Grant et al., 2006; Squeglia et al., 2009; Patte et al., 2017). Early initiation of substance use is also prospectively associated with experiencing more negative consequences (Marino and Fromme, 2016). Thus, identifying factors that are associated with increased impulsivity among adolescents is of critical importance.

One factor that has been associated with increased impulsivity and substance use outcomes among adolescents is the mental health of their parents, including psychopathology (Zhang et al., 2020) and maternal depressive symptoms (Felton et al., 2021). This finding aligns with substantial evidence which has linked parental mental health conditions with various deleterious outcomes for their children, including higher levels of internalizing, externalizing, and general psychopathology (Goodman et al., 2011). Internalizing problems are characterized by anxiety and depressive symptoms, whereas externalizing problems are characterized by aggressive and impulsive symptoms (Achenbach et al., 2016; Nikstat and Riemann, 2020). However, ways in which parental mental health conditions increase their children’s impulsivity are less well understood. Identifying factors that link parental mental health (e.g., internalizing and externalizing problems) and their children’s impulsivity could be informative in identifying intervention targets and developing tailored interventions that could, in turn, potentially reduce the risk for early substance use.

Whereas some research has examined the role of genetic and biological factors to explain relations between parental mental health conditions and their children’s health outcomes (Rende and Plomin, 1993; Kim-Cohen, 2007), substantial research has also examined environmental mechanisms by which parental mental health conditions affect their children’s outcomes (Cummings and Davies, 2006; Natsuaki et al., 2014), with a particular focus on parenting and the parent–child relationship as a social context for youth development. For example, more depressive symptoms among mothers has been associated with more critical comments to their children (Webster-Stratton and Hammond, 1988). Neglect also mediates relations between mother’s history of depression and child’s psychiatric disorder (Bifulco et al., 2002). Meta-analytic evidence shows that maternal depression is associated with negative maternal behavior (including hostile or coercive behavior by the mother), but not disengaged or positive behaviors (Lovejoy et al., 2000).

Parental mental health conditions are also associated with increased family conflict (Chang et al., 2001; Sarigiani et al., 2003). Maternal depression has been shown to affect the positive and negative emotions of their adolescent children, a relationship that was mediated by family conflict and emotional expressiveness (Yeh et al., 2016). Family environment (i.e., cohesion, expressiveness, and conflict) also mediates relations between parental and adolescent mental health conditions (Van Loon et al., 2014). Family conflict has been associated with more deleterious outcomes in adolescents and adults (Rothenberg et al., 2017). Recent work also shows that family conflict mediates relations between impulsivity and early substance use (Wang et al., 2021), whereas others, using cross sectional data, have also found that impulsivity mediates relations between family conflict and tobacco use among adolescents (Eslava et al., 2022). Further, the majority of the literature on the impact of parental mental health on children’s health outcomes has largely focused on maternal depressive symptoms, which has resulted in a “mother-bashing” quality to the literature (Downey and Coyne, 1990), providing an incomplete picture of family dynamics (Ramchandani and Psychogiou, 2009; Pierce et al., 2020).

Taken together, evidence suggests that family conflict and impulsivity are linked with substance use outcomes. However, the hypothesis that parental mental health conditions may be related to increased family conflict which in turn may be related to increased impulsivity among their children has, to our knowledge, yet to be tested in a large sample and using prospective data. Specifically, from a public health perspective, efforts to promote family cohesion and reduce family conflict may serve as intervention targets to alleviate increased impulsivity among adolescents, and potentially resulting substance use.

To address these gaps, the current report used data from the large national Adolescent Brain Cognitive Development (ABCD) study to examine whether family conflict mediates relations between parental mental health (i.e., internalizing, externalizing, and total problems) and adolescent impulsivity. The ABCD study is the largest single-cohort prospective longitudinal study of children’s health and neurodevelopment in the United States (US), following the lives of 11,880 children and their parents/guardians for at least 10 years (Barch et al., 2018; Garavan et al., 2018). ABCD conducts in-person assessments on an annual basis (i.e., waves), and was designed to approximate the US in key sociodemographic variables (e.g., sex, race, household income, parental education; Dick et al., 2021).

**Figure 1:**
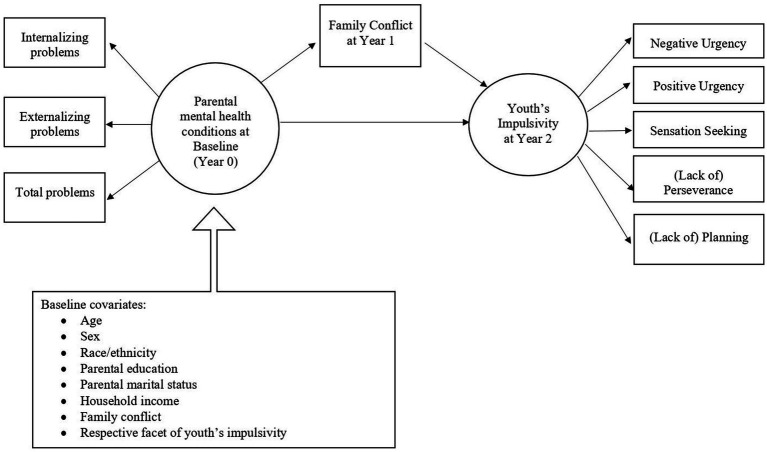
Conceptual model of family conflict mediation effects between parental mental health and youth’s impulsivity.

The conceptual model for the study is presented in Figure 1. Specifically, we hypothesized that family conflict at year 1 would mediate relations between parental mental health conditions at baseline and youth’s impulsivity at year 2. Using data from the ABCD study to examine this hypothesis is particularly advantageous for two reasons. First, evidence suggests that detecting mediation effects, which are typically small relative to direct effects, requires more power (Dick et al., 2021), underscoring the value of the large sample in the ABCD data. Second, the longitudinal design of the ABCD study allows for temporal ordering of study variables, giving more insight into possible causal mechanisms, and extending previous cross-sectional analyses.

## 2. Materials and methods

### 2.1. Participants and procedure

Data were from the first three annual waves of the ABCD study data collection (Data Release 4.0). Study design and participant demographics have been previously described in detail elsewhere (Barch et al., 2018; Garavan et al., 2018). At baseline, there were 11,876 children, who were between ages 9 and 10 years. Baseline data were collected between September 2016 and November 2018; and follow-up visits were conducted at yearly intervals.

### 2.2. Measures

**Exposure: Parental mental health conditions**. At baseline (year 0), parents completed the Achenbach System of Empirically Based Assessment (ASEBA) adult self-report (ASR; Achenbach, 2019). Parents reported on their own emotional/behavior problems and their own mental health status. Parents rated 120 problem items on a scale of 0 (*Not True*) to 2 (*Very True or Often True*) for the previous 6 months. The measure is designed to assess dimensional psychopathology (Achenbach, 2019); the items correspond with DSM-oriented and empirically-derived syndrome scales, with composite scores for internalizing and externalizing psychopathology. The ASR also provides a total score for parental mental health and related problems (Middeldorp et al., 2016). The ABCD study also makes available T-scores for each composite score of internalizing, externalizing, and total problems, which are normed for each gender by age based on national probability samples (Albar and Sattar, 2022). For each composite score, a score of 70 or above are considered within the clinical range for psychopathology symptoms and behavioral dysfunction, whereas scores of 64 or below are considered in the normal range (Achenbach, 2015, 2019). Although the self-report is not a direct measure of DSM-5 symptoms, scores on the measure are associated with DSM diagnoses (Hofstra et al., 2001, 2002). T-scores were used in the mediation analyses to aid in interpretability and comparison with other studies (e.g., Albar and Sattar, 2022).

**Mediator: Family conflict**. Adolescents completed the Family Conflict subscale of the Family Environment Scale (Zucker et al., 2018) at year 1, which was used as the mediator variable. The nine-item true/false scale included items such as “Family members often criticize each other,” “We fight a lot in our family,” and “Family members sometimes get so angry they throw things.” A family conflict index was computed by summing responses.

**Outcome: Youth impulsivity**. Adolescents’ self-reported impulsivity was measured via the 20-item Urgency, Planning (lack of), Perseverance (lack of), Sensation Seeking, Positive Urgency, Impulsive Behavior (UPPS-P) scale (Barch et al., 2018). The UPPS-P completed at year 2 follow-up was used as the outcome variable.

**Covariates:** Covariates were participants’ demographic characteristics (i.e., age, sex, race, household income, parental education, and parental marital status). We also adjusted for baseline levels of family conflict and UPPS-P subscales.

### 2.3. Statistical analyses

Parental mental health (exposure) and family conflict (mediator) were measured at all three time points, whereas impulsivity (outcome) was measured at baseline and year 2 only. T-scores of parental mental health conditions were used to facilitate interpretation of results. We conducted longitudinal (i.e., lagged) mediation models, which used standardized versions of parental mental health at baseline as the exposure, family conflict at year 1 as mediator, and UPPS scores at Year 2 as outcomes. Longitudinal models maintain the temporal precedence needed to examine mediation analyses. Longitudinal models also controlled for baseline covariates, including sex, age, race, household income, parental education and marital status, as well as the values of the mediator (i.e., family conflict) and the outcome (i.e., the respective facet of impulsivity) variables, which were added as fixed effects. A random intercept for family ID was also included in mediation models to address clustering due to the presence of siblings in the ABCD data. Each model used data from participants with complete data across the composite variables for the specific set of analyses, which resulted in a final sample of *N* = 9,657 for longitudinal analyses. Multi-level causal mediation analyses were conducted in R (version 4.2.2), using the *mediation* package (Tingley et al., 2014). Variance explained for the total effect was determined by calculating *R^2^* via the *r.squaredGLMM* function in the MuMIn package (Nakagawa et al., 2017). In line with previous recommendations, both marginal *R^2^* (i.e., variance explained by only fixed effects) and conditional *R^2^* (i.e., variance explained by both fixed and random effects) are reported (Nakagawa and Schielzeth, 2013).

## 3. Results

Table 1 shows sample characteristics. At baseline, youth were, on average, 9.92 years old (*SD* = 0.62), 10.92 years old (*SD* = 0.64) at year 1, and 12 years old (*SD* = 0.66) at year 2. The sample was 52% White, 20% Hispanic, 15% Black, 2% Asian, and 11% other race/ethnicity. The majority of the sample was from households that were married (67%), college educated or above (53%), and with a household income of over $75,000 (58%). The ABCD study sample was recruited via methods that sought to reduce selection bias, but the resulting data are not meant to be representative of all US youth (Garavan et al., 2018). Although the sample is diverse and generally mirrors the US population in race/ethnicity characteristics, the sample is also more educated, has more children from married caregivers, and higher income than the average US household (Gard et al., 2023).

**Table 1:** Sample characteristics.

Sample characteristics	Frequency (Percent) at baseline *N* = 11,876
Sex (Female)	5,680 (47.8)
Race/ethnicity	
White	6,176 (52.1)
Hispanic	2,404 (20.3)
Black	1,784 (15)
Asian	252 (2.1)
Other	1,247 (10.5)
Parental marital status	
Married	7,982 (67)
Never married	1,458 (12)
Divorced	1,080 (9)
Living with partner	688 (6)
Parent education	
Less than high school	785 (6.6)
High school or GED	1,259 (10.6)
Some college/AA	3,483 (29.4)
College degree	3,330 (28.1)
Master’s or higher	2,991 (25.2)
Income	
< $25,000	1,643 (15.1)
$25,000–$50,000	1,586 (14.6)
$50,000–$74,999	1,498 (12.8)
$75,000–$99,999	1,570 (14.5)
$100,000–$199,999	3,313 (30.5)
$200,000 <	1,249 (11.5)

Descriptive statistics and correlations are reported in Table 2. Parent’s average self-rated mental health scores were in the normative range for internalizing (Mean T-score = 48.09; *SD* = 10.61; range = 0–70), externalizing (Mean T-score = 45.93; *SD* = 9.64; range = 0–90), and total problems (Mean T-score = 42.99; *SD* = 10.23; range = 25–89). Family conflict scores were, on average, 2.04 (*SD* = 1.95; range: 0–9) at baseline and 1.92 (*SD* = 1.88) at year 1 follow-up. All study variables were significantly related to each other, except lack of significant relations between parental mental health conditions and sensation seeking.

**Table 2:** Descriptive statistics and bivariate correlations.

	Mean (SD)	1	2	3	4	5	6	7	8	9	10	11	12	13	14	15
Baseline (*N* = 11,876)																
P Inter. prob.	9.53 (8.74)	–														
P Exter. prob.	5.55 (5.65)	0.62**	–													
P Total prob.	21.12 (17.96)	0.9**	0.82**	–												
Y Fam conft	2.04 (1.95)	0.09**	0.12**	0.11**	–											
Y Negative urgency	8.49 (2.65)	0.04**	0.05**	0.05**	0.25**	–										
Y Positive urgency	7.99 (2.96)	0.06**	0.07**	0.07**	0.24**	0.49**	–									
Y Lack of planning	7.74 (2.38)	0.04**	0.06**	0.06**	0.2**	0.16**	0.21**	–								
Y Lack of perseverance	7.04 (2.25)	0.08**	0.07**	0.09**	0.17**	0.13**	0.17**	0.45**	–							
Y Sensation seeking	9.77 (2.68)	−0.01	0.004	0.002	0.02*	0.14*	0.19**	0.06**	−0.1**	–						
Year 1 (*N* = 11,225)																
Y Family conflict	1.92 (1.88)	0.1**	0.14**	0.13**	0.46**	0.18***	0.18***	0.17***	0.15***	0.002	–					
Year 2 (*N* = 10,414)																
Y Negative urgency	7.76 (2.34)	0.08**	0.09**	0.09**	0.17***	0.29**	0.25***	0.14***	0.11***	0.06***	0.21**	–				
Y Positive urgency	7.39 (2.67)	0.07**	0.09**	0.09**	0.16***	0.2***	0.32***	0.13***	0.13***	0.08***	0.2**	0.54**	–			
Y Lack of planning	7.77 (2.24)	0.07**	0.09**	0.09**	0.14***	0.16***	0.18***	0.39***	0.25***	0.09***	0.2**	0.28**	0.28**	–		
Y Lack of perseverance	6.97 (2.25)	0.1**	0.1**	0.12**	0.17***	0.14***	0.16***	0.25***	0.4***	−0.03**	0.2**	0.24**	0.24**	0.47**	–	
Y Sensation seeking	9.48 (2.68)	−0.01	0.02	0.01	−0.002	0.04***	0.07***	0.09***	−0.04**	0.43**	0.00*	0.18**	0.24**	0.14**	−0.07**	–

Notes: P, Parent. Y, Youth. Inter. prob., Internalizing problems. Exter. prob., Externalizing problems. Total prob., Total problems. Internalizing, externalizing, and total problems were log-transformed to reduce skew and kurtosis. *p < 0.05; **p < 0.01; ***p < 0.001.

Tables 3–5 show results from the longitudinal mediation models, each using internalizing, externalizing, and total parental problems as the predictor variable, adjusting for covariates at baseline, respectively.

**Table 3:** Longitudinal mediation effects of family conflict at year 1 on relations between internalizing problems at baseline and children’s impulsivity at year 2.

	Direct effect^a^	Indirect effect^b^	Total effect^c^	Mediated proportion by family conflict^d^	*p*-value^e^	*R^2^M (R^2^C)*
Negative urgency	*β* = 0.05 [0.03, 0.07]	*β* = 0.007 [0.005, 0.01]	*β* = 0.06 [0.04, 0.08]	*β* = 0.13 [0.08, 0.22]	<0.001***	0.006 (0.21)
Positive urgency	*β* = 0.04 [0.02, 0.06]	*β* = 0.007 [0.004, 0.01]	*β* = 0.04 [0.03, 0.06]	*β* = 0.16 [0.09, 0.28]	<0.001***	0.005 (0.15)
Lack of planning	*β* = 0.04 [0.02, 0.06]	*β* = 0.007 [0.005, 0.01]	*β* = 0.05 [0.03, 0.06]	*β* = 0.15 [0.09, 0.28]	<0.001***	0.005 (0.11)
Lack of perseverance	*β* = 0.05 [0.04, 0.07]	*β* = 0.005 [0.003, 0.01]	*β* = 0.06 [0.04, 0.08]	*β* = 0.9 [0.05, 0.14]	<0.001***	0.01 (0.19)
Sensation seeking	*β* = −0.001 [−0.02, 0.02]	*β* = 0.0009 [−0.00003, 0.00]	*β* = −0.0004 [−0.02, 0.02]	*β* = −0.01 [−1.89, 2.06]	0.96	–

*N* = 9,657 with complete data. Each cell reports estimate betas with 95% confidence intervals. All models included random effect of family identified and controlled for covariates (age, sex, race, parental education, parental marital status, and income), baseline levels of family conflict (mediator) and baseline levels of respective impulsivity facet (outcome). *R^2^M*, Marginal *R^2^*. R^2^C, Conditional *R^2^*. ^a^Parental internalizing problems to child’s impulsivity. ^b^Parental internalizing problems to child’s impulsivity via family conflict. ^c^Sum of direct and indirect effects. ^d^Indirect effect *β* divided by total effect *β*. ^e^*p* value reported is for effect of proportion mediation. *** *p* < 0.001.

**Table 4:** Longitudinal mediation effects of family conflict at year 1 on relations between externalizing problems at baseline and children’s impulsivity at year 2.

	Direct effect^a^	Indirect effect^b^	Total effect^c^	Mediated proportion by family conflict^d^	*p*-value^e^	*R^2^M (R^2^C)*
Negative urgency	*β* = 0.04 [0.02, 0.06]	*β* = 0.01 [0.008, 0.01]	*β* = 0.05 [0.03, 0.07]	*β* = 0.20 [0.13, 0.34]	<0.001***	0.007 (0.21)
Positive urgency	*β* = 0.05 [0.03, 0.07]	*β* = 0.01 [0.008, 0.01]	*β* = 0.06 [0.04, 0.08]	*β* = 0.19 [0.12, 0.32]	<0.001***	0.007 (0.15)
Lack of planning	*β* = 0.04 [0.02, 0.05]	*β* = 0.009 [0.007, 0.01]	*β* = 0.05 [0.03, 0.06]	*β* = 0.21 [0.13, 0.37]	<0.001***	0.006 (0.12)
Lack of perseverance	*β* = 0.05 [0.03, 0.07]	*β* = 0.007 [0.005, 0.01]	*β* = 0.06 [0.04, 0.07]	*β* = 0.13 [0.08, 0.2]	<0.001***	0.01 (0.2)
Sensation seeking	*β* = 0.05 [−0.07, 0.03]	*β* = 0.001 [−0.0006, 0.00]	*β* = 0.02 [0.002, 0.04]	*β* = 0.05 [−0.07, 0.31]	0.26	–

*N* = 9,657 with complete data. Each cell reports estimate betas with 95% confidence intervals. All models included random effect of family identified and controlled for covariates (age, sex, race, parental education, parental marital status, and income), baseline levels of family conflict (mediator) and baseline levels of respective impulsivity facet (outcome). *R^2^M*, Marginal R^2^. *R^2^C*, Conditional *R^2^*. ^a^Parental internalizing problems to child’s impulsivity. ^b^Parental internalizing problems to child’s impulsivity via family conflict. ^c^Sum of direct and indirect effects. ^d^Indirect effect *β* divided by total effect *β*. ^e^*p* value reported is for effect of proportion mediation. ** *p* < 0.001.

**Table 5:** Longitudinal mediation effects of family conflict at year 1 on relations between total problems at baseline and children’s impulsivity at year 2.

	Direct effect^a^	Indirect effect^b^	Total effect^c^	Mediated proportion by family conflict^d^	*p*-value^e^	*R^2^M (R^2^C)*
Negative urgency	*β* = 0.06 [0.04, 0.08]	*β* = 0.009 [0.007, 0.01]	*β* = 0.07 [0.05, 0.09]	*β* = 0.14 [0.09, 0.21]	<0.001***	0.009 (0.21)
Positive urgency	*β* = 0.05 [0.03, 0.07]	*β* = 0.009 [0.006, 0.01]	*β* = 0.06 [0.04, 0.08]	*β* = 0.16 [0.1, 0.25]	<0.001***	0.007 (15)
Lack of planning	*β* = 0.04 [0.02, 0.06]	*β* = 0.009 [0.006, 0.01]	*β* = 0.05 [0.03, 0.07]	*β* = 0.17 [0.11, 0.29]	<0.001***	0.007 (0.12)
Lack of perseverance	*β* = 0.07 [0.05, 0.08]	*β* = 0.006 [0.004, 0.01]	*β* = 0.07 [0.05, 0.09]	*β* = 0.9 [0.06, 0.14]	<0.001***	0.015 (0.2)
Sensation seeking	*β* = 0.007 [−0.001, 0.03]	*β* = 0.0009 [−0.0005, 0.00]	*β* = 0.008 [−0.01, 0.03]	*β* = 0.06 [−1.07, 1.01]	0.54	–

Note: *N* = 9,657 with complete data. Each cell reports estimate betas with 95% confidence intervals. All models included random effect of family identified and controlled for covariates (age, sex, race, parental education, parental marital status, and income), baseline levels of family conflict (mediator) and baseline levels of respective impulsivity facet (outcome). *R^2^M*, Marginal *R^2^*. *R^2^C*, Conditional *R^2^*. ^a^Parental internalizing problems to child’s impulsivity. ^b^Parental internalizing problems to child’s impulsivity via family conflict. ^c^Sum of direct and indirect effects. ^d^Indirect effect *β* divided by total effect *β*. ^e^*p* value reported is for effect of proportion mediation. *** *p* < 0.001.

Across all models, there were small significant direct effects of parental mental health conditions at baseline on youth’s impulsivity at year 2, except sensation seeking. For internalizing problems, direct effects (i.e., standardized *β*) were 0.05 for negative urgency and lack of perseverance and 0.04 for positive urgency and lack of planning. In other words, for instance, using 1 standard deviation as a marker, which would be 10 T-score points, as T-score for internalizing problems increases by 10, a 0.5 standard deviation increase is observed in negative urgency. Direct effects of externalizing problems were similar, at 0.05 for positive urgency and lack of perseverance; and 0.04 for negative urgency and lack of planning. Direct effects of total problems on youth’s impulsivity were slightly higher, ranging from 0.04 for lack of planning to 0.07 for lack of perseverance.

Across models, results showed that family conflict at year 1 significantly mediated relations between each of the three parental mental health conditions at baseline and each facet of impulsivity at year 2, except sensation seeking. That is, all models revealed an indirect effect of baseline parental mental health conditions on facets of youth impulsivity (except sensation seeking) at year 2 via family conflict at year 1.

The proportion mediated for the associations between internalizing problems and each facet of youth impulsivity via family conflict ranged between 9% (for lack of perseverance) to 16% (for positive urgency). The proportion mediated for the effect of externalizing problems on youth impulsivity via family conflict was slightly higher, ranged from 13% (for lack of perseverance) to 21% (for lack of planning). The proportion mediated for total problems was similar to internalizing problems.

Marginal *R^2^* effect sizes indicated a very small amount of the variance is explained by the fixed direct effects of each of the three parental mental health conditions on facets of youth’s impulsivity, which ranged between 0.005 to 0.015. Conditional *R^2^* effect sizes indicate that the variance explained by the entire model (i.e., fixed and random effects) was larger than the marginal *R^2^*.

## 4. Discussion

The current study used data from the ABCD study to examine the mediating role of family conflict in relations between parental mental health conditions and youth impulsivity. Results using longitudinal data, which maintain the temporal sequence of study variables as hypothesized, indicate that within-participant changes in the level of family conflict between baseline and year 1 explained changes in the strength of relations between baseline parental mental health and year 2 youth impulsivity. Identifying potential pathways through which parental mental health conditions may impact youth’s impulsivity and the extent to which family conflict mediates these relations, particularly using a large national dataset and prospective data, advances the existing literature and informs efforts aimed at reducing youth’s impulsivity and family conflict.

Findings are in line with literature that suggests that paternal mental health conditions may create challenges in parent–child relationships (Downey and Coyne, 1990; Goodman et al., 2011; Natsuaki et al., 2014) and aligned with evidence that shows family conflict is associated with children’s impulsivity (Elam et al., 2016). Current results suggest that difficulties with family functioning (i.e., increased family conflict) are an important social context in youth development, and may be one intergenerational mechanism linking parental mental health and youth’s impulsivity. Effective family interventions (e.g., family psychoeducation, skills training programs) may be indicated for those with high family conflict.

Although mediation findings were significant in the expected direction, and family conflict explained a not insignificant amount of the effect of parental mental health on youth’s impulsivity, it is worth noting that observed effect sizes were quite small. If true effects of parental mental health and family conflict on youth’s impulsivity are indeed as small as results indicate, it could be the case that significant mediation relations may not have been observed in smaller samples, highlighting one unique advantage of the large ABCD study. Because the prospective (i.e., lagged) mediation relations between study variables has not been previously examined in comparable ways, it is challenging to compare observed effect sizes with the literature. However, research shows smaller sample sizes are associated with higher effect sizes (Cortina and Landis, 2009; Zhang et al., 2013; Bakker et al., 2019). Further, publication bias of research studies is also related to increased effect sizes (Yang et al., 2023). Thus, it could be the case that the previously reported effect size of relations between parental mental health, family conflict, and youth’s impulsivity may be larger than their true relationship, in part due to smaller sample sizes. Although caution is advised in over-interpreting findings from the current study, research shows that small effect sizes can also have practical or clinical utility (Dick et al., 2021). In line with current results, others using ABCD data have also found very small effect sizes (Fahey et al., 2023). The extent to which how much improvement would occur in youth’s impulsivity, if family conflict were reduced or parental mental health problems were treated is an important research question that requires empirical examination. Results may also have been impacted by reporter effects, such that observed effects may have been stronger with a parent report of family conflict, which may have been more highly correlated with parent reports of mental health conditions. Future research can also address this by examining relations between study variables using multiple reporters.

Current findings can also be contextualized within the extant literature using ABCD data. Others using the ABCD data have also found that genetic risk, using polygenic scores, and family conflict are associated with higher impulsivity (Su et al., 2022). Fahey et al. (2023), using ABCD data, found that impulsivity in children was related to externalizing behaviors, supporting the importance of targeting impulsivity. Leveraging the unique neuroimaging data available in the ABCD study, other researchers have also found that relations between family conflict and children’s outcomes is mediated by brain structure (Gong et al., 2021; Teeuw et al., 2023). Taken together, the ABCD study presents an ideal opportunity to further examine ways in which parental mental health may increase youth’s impulsivity, in part via family and neural mechanisms.

Although study variables were generally significantly associated as expected, the sensation-seeking domain in the UPPS measure was consistently not associated with parental mental health conditions or family conflict. Others using ABCD data (e.g., Wang et al., 2021) have also found that sub-traits of impulsivity are differentially related to outcomes. Specifically, Wang et al. (2021) found that, compared to youth who never had a puff of tobacco, those who had a puff of tobacco had higher impulsivity across all facets except sensation seeking. Other evidence also suggests that facets of impulsivity may be differentially related to various substance use outcomes (Adams et al., 2012). Further, sensation seeking does not conform to the Moeller et al. (2001) definition of impulsivity because those with high sensation seeking do not necessarily act with diminished regard for possible negative consequences. Results from the current report showing effects of parental mental health and family conflict on all facets of impulsivity except sensation seeking are in line with previous research, and indicate potentially unique pathways leading to different impulsive traits.

The study has several limitations. Although this research focuses on how parental and family factors shape youth’s impulsivity, research shows there may be reciprocal relations, such that youth’s impulsivity may also contribute to increased family conflict, leading to early substance use (Wang et al., 2021). The present analyses statistically controlled for family conflict and youth’s impulsivity at baseline (i.e., entered as a covariate) when predicting youth’s impulsivity at year 2. Nevertheless, future research can leverage the ABCD Study to examine cross-lagged relations and possibly disentangle the direction of prospective influences among these variables. Study measures also relied on self-report, which are open to recall and desirability biases, which can be addressed in future studies, including by using standard cognitive tasks and functional magnetic resonance imaging (fMRI) data. Further, the family conflict scale did not include a specified window of assessment (e.g., past month, past year), which can be addressed in future research. As study participants get older (and have more substance use exposure), we will be able to examine how current findings impact future substance use.

Taken together, results indicate that family conflict is an important social influence for youth development, mediating the effect of parental mental health conditions on youth’s impulsivity. Family-based interventions to reduce conflict may have the potential to buffer the effects of parental mental health conditions on youth’s impulsivity and, in turn, substance use outcomes. Evidence suggests family-based interventions (e.g., brief family therapy), particularly youth-focused intervention components (e.g., positive family relations), are efficacious in preventing substance misuse (Kumpfer et al., 2003; Van Ryzin et al., 2016). Research in other areas also indicates that family-based psychosocial interventions are efficacious in addressing family functioning (O’Donnell et al., 2020), which can be tested in future interventions aimed at adolescent impulsivity and family conflict.

## Data availability statement

Publicly available datasets were analyzed in this study. This data can be found at: https://osf.io/8vpm9/?view_only=e546d0e2c65b46cb9f8db4844888991e.

## Author contributions

NG conceptualized the study with input from all authors. NG and NA organized the dataset. NG and WT performed the statistical analysis. All authors contributed to the article and approved the submitted version.
